# Supplementary material for: Regular Training Increases sTWEAK and Its Decoy Receptor sCD163–Does Training Trigger the sTWEAK/sCD163-Axis to Induce an Anti-Inflammatory Effect?
Source: J Clin Med. 2020 Jun 17;9(6):1899. doi: 10.3390/jcm9061899 (PMC7356003; doi:10.3390/jcm9061899)
Supplement: Supplementary file 1 [file jcm-09-01899-s001.pdf]

	sCD163		sTWEAK	
	Corr.coeff.	*p*-value	Corr.coeff.	*p*-value
Erythrocytes	0.237	0.020	0.265	0.009
Hemoglobin			0.210	0.039
Hematocrit	0.211	0.038	0.240	0.018
Calcium			0.250	0.013
Uric acid	0.246	0.015	0.275	0.006
Calcitonine			0.257	0.011
Testosterone			0.210	0.039
BMI	0.337	0.001		
Body water	−0.241	0.017		
Body fat	0.260	0.010		
ASAT	0.408	<0.001		
ALAT	0.412	<0.001		
Gamma GT	0.315	0.002		
LDH	0.259	0.011		
Lipoprotein a	0.224	0.027		
hsCRP	0.207	0.042		
Interleukin-6	0.338	0.001		

Table S1. Significant results of the Spearman correlation using the data set of the whole population at baseline. In a second step, all significant correlations of the Spearman test were included in a linear regression model with backwards method (see Table 2).

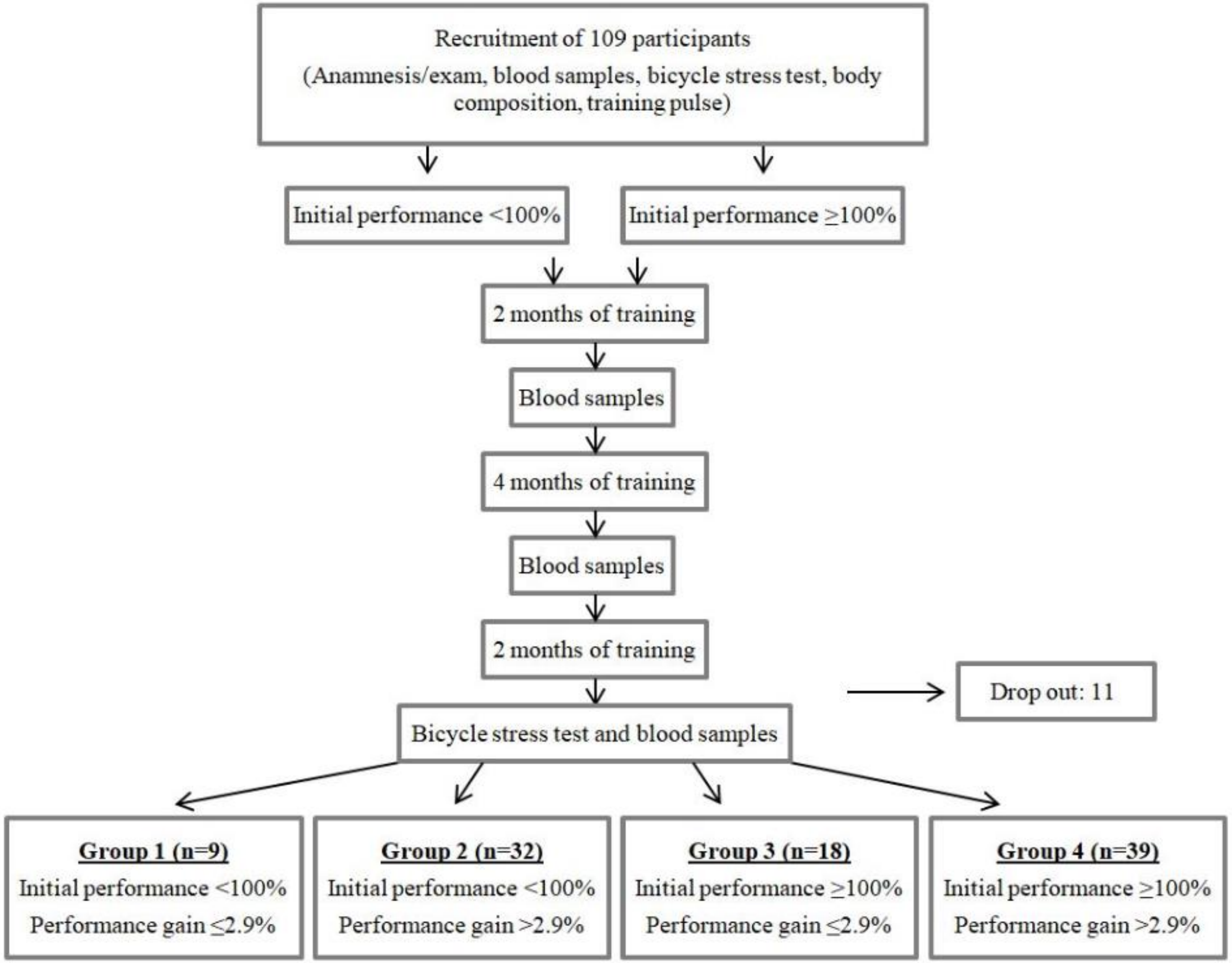

Figure S1: Recruitment and study procedure.
